# PiT2 deficiency prevents increase of bone marrow adipose tissue during skeletal maturation but not in OVX-induced osteoporosis

**DOI:** 10.3389/fendo.2022.921073

**Published:** 2022-11-16

**Authors:** Giulia Frangi, Marie Guicheteau, Frederic Jacquot, Grzegorz Pyka, Greet Kerckhofs, Magalie Feyeux, Joëlle Veziers, Pierre Guihard, Boris Halgand, Sophie Sourice, Jérôme Guicheux, Xavier Prieur, Laurent Beck, Sarah Beck-Cormier

**Affiliations:** ^1^ Nantes Université, Oniris, CHU Nantes, Inserm, Regenerative Medicine and Skeleton, RMeS, UMR 1229, SFR Bonamy, Nantes, France; ^2^ Nantes Université, CHU Nantes, Inserm, CNRS, CRCI2NA, Nantes, France; ^3^ Biomechanics lab, Institute of Mechanics, Materials, and Civil Engineering, UC Louvain, Louvain-la-Neuve, Belgium; ^4^ Department of Materials Engineering, KU Leuven, Leuven, Belgium; ^5^ IREC, Institute of Experimental and Clinical Research, UC Louvain, Woluwé-Saint-Lambert, Belgium; ^6^ Prometheus, Division of Skeletal Tissue Engineering, KU Leuven, Leuven, Belgium; ^7^ Nantes Université, CHU Nantes, CNRS, Inserm, BioCore, US16, SFR Bonamy, Nantes, France; ^8^ Nantes Université, CNRS, Inserm, l’Institut du Thorax, Nantes, France

**Keywords:** PiT2/Slc20a2, adipocytes, bone, post-menopausal osteoporotic mouse model, marrow

## Abstract

The common cellular origin between bone marrow adipocytes (BMAds) and osteoblasts contributes to the intimate link between bone marrow adipose tissue (BMAT) and skeletal health. An imbalance between the differentiation ability of BMSCs towards one of the two lineages occurs in conditions like aging or osteoporosis, where bone mass is decreased. Recently, we showed that the sodium-phosphate co-transporter PiT2/SLC20A2 is an important determinant for bone mineralization, strength and quality. Since bone mass is reduced in homozygous mutant mice, we investigated in this study whether the BMAT was also affected in *PiT2^-/-^* mice by assessing the effect of the absence of PiT2 on BMAT volume between 3 and 16 weeks, as well as in an ovariectomy-induced bone loss model. Here we show that the absence of PiT2 in juveniles leads to an increase in the BMAT that does not originate from an increased adipogenic differentiation of bone marrow stromal cells. We show that although *PiT2^-/-^* mice have higher BMAT volume than control *PiT2^+/+^* mice at 3 weeks of age, BMAT volume do not increase from 3 to 16 weeks of age, leading to a lower BMAT volume in 16-week-old *PiT2^-/-^* compared to *PiT2^+/+^* mice. In contrast, the absence of PiT2 does not prevent the increase in BMAT volume in a model of ovariectomy-induced bone loss. Our data identify *SLC20a2/PiT2* as a novel gene essential for the maintenance of the BMAd pool in adult mice, involving mechanisms of action that remain to be elucidated, but which appear to be independent of the balance between osteoblastic and adipogenic differentiation of BMSCs.

## Introduction

Bone marrow adipose tissue (BMAT) is no longer considered a marrow space filler tissue as the increasing number of studies on its function in the past 20 years has highlighted its important roles in skeletal health or energetic metabolism. However, the underlying cellular and molecular mechanisms involved remain to be determined (1–3). In mice, bone marrow adipocytes (BMAds) first develop in the distal tibia at a very early postnatal age and then appear in the proximal tibia at around 3 months of age (3, 4). The volume of BMAT increases with age in humans and mice, turning the bone marrow from “red” to “yellow”. The proximal and distal BMAT are two distinct tissues mainly because the size and the lipidic components of the adipocytes are different, and both are differentially regulated (5). The proximal BMAT is also referred to as regulated BMAT (rBMAT) due to the observation that its volume is regulated by several physiopathological conditions (caloric restriction, cold exposure…), whereas the distal one is referred to as constitutive BMAT (cBMAT) because volume variations of this tissue are less frequently observed.

A growing interest in BMAT and its intimate relationship with skeletal health has emerged in recent years. Inverse correlation between BMAT volume and bone mass has been described in many situations (5) and also in some genetic mouse models (6–11). However, this negative correlation is not always observed and may even be controversial, as in the ovariectomy-induced osteoporosis model (7, 11). In healthy populations, BMAT increases with age both in men and women, with men generally having more BMAT (12, 13), and an inverse correlation between BMAT volume and bone mineral density (BMD) has been described (14–17). Nevertheless, during puberty, the amount of marrow fat is positively associated with total bone mineral content in girls (18) and, although men have more BMAT mass compared to women, they also have more bone mass (19). These observations highlight a complex relationship between BMAT and bone, which probably cannot be described in all circumstances as a simple inverse correlation.

Nonetheless, the relationship between BMAT and bone is based at least on the fact that osteoblasts and BMAds are both derived from bone marrow stromal cells (BMSCs). The imbalance between the differentiation capacities of BMSCs towards one of the two lineages is at the origin of the increased bone marrow adiposity in conditions like aging, obesity and other pathologies where the bone mass is decreased (11, 20, 21) and a better knowledge of the underlying mechanisms would allow a better understanding of the balancing relationship between bone and BMAT.

PiT2 is a member of the Slc20 family of sodium (Na)-phosphate (Pi) co-transporters, encoded by the *Slc20a2* gene. In the last 10 years, numerous studies have revealed the multifunctionality of PiT1/SLC20A1 and PiT2/SLC20A2 in the regulation of cellular proliferation, differentiation or survival (22–30). In bone, our group and others have shown the involvement of PiT2 in bone quality, mineralization and strength (31, 32). Homozygous mutant mice were shown to exhibit severe reduction of bone mechanical properties (decreased yield load, maximum load, fracture load and stiffness), reduced bone mineral density and reduced bone mass (31, 32). Considering the deleterious effect of PiT2 deficiency in bone, we questioned whether the BMAT could also be affected in PiT2 knockout mice. In addition, we evaluated the effect of the absence of PiT2 on the regulation of bone and BMAT volumes between 3 and 16 weeks and in a model of ovariectomy-induced bone loss.

## Materials & methods

### Mice

C57BL/6NTac-Slc20a2tm11a (EUCOMM) Wtsi (*Slc20a2^+/-^*, hereafter named *PiT2^+/-^*) heterozygous mice were obtained from the European Mouse Mutant Archive (EMMA) and maintained and genotyped at Nantes Université. Experiments on mice were conducted according to the French and European regulations on care and protection of laboratory animals (EC Directive 86/609, French Law 2001-486 issued on June 6, 2001). This study complied with ARRIVE (Animal Research: Reporting of *In Vivo* Experiments) guidelines and was approved by the Animal Care Committee of Pays de la Loire (APAFIS agreements 02286.02, 14835-2020-05-13 and 22178-2019092614593965 v7). Animal care and maintenance were provided through the Nantes Université accredited animal facility at the “Unité de Thérapeutique Expérimentale” (UTE). Mice were housed under specific pathogen-free conditions in open or individually ventilated cages, with wood shavings for bedding and nesting material, in groups of up to five. The mice had *ad libitum* access to tap water and standard rodent chow (A04-10; SAFE, France). Genotyping was performed by PCR as described (31). Wild-type (*PiT2^+/+^*) and *PiT2^-/-^* littermate mice were analysed at 3 and 16 weeks. Three-week-old mice were used for *in vitro* experiments (adipogenic differentiation, flow cytometry). Ovariectomy (OVX) was performed under isoflurane anesthesia in 13-week-old female *PiT2^+/+^* and *PiT2^-/-^* littermate mice, which were euthanized 5 weeks later. *PiT2^+/+^* and *PiT2^-/-^* mice were randomly allocated to two groups: an OVX group and a sham group (mice were similarly incised but the ovaries were not removed). Mice were anesthetized with ketamine/xylazine administered intraperitoneally and were perfused transcardially with Phosphate Buffered Saline (PBS) and then 4% Paraformaldehyde (PFA; Sigma Aldrich, USA). Uterine aplasia was observed, confirming the successful ovariectomy. Right tibiae were removed and fixed in 4% PFA for 24 hours at 4°C and stored in PBS at 4°C for the subsequent CE-CT analyses.

### Contrast-enhanced high resolution microfocus computed tomography

Before polyoxometalate-staining (33), the distal end of the bones was removed to allow better diffusion of the contrast agent into the bone marrow compartment. Samples were incubated in the staining solution during 48 hours at 4°C while shaking gently.

CE-CT acquisition and image processing were performed as previously described (33, 34). Briefly, samples were imaged using a Phoenix Nanotom S (GE Measurement and Control Solutions, Germany) and analysed using DataViewer (Bruker MicroCT, Belgium) for the reorientation of the CE-CT datasets and CTAn (Bruker MicroCT) for the assessment of the fat volume in the proximal and distal tibiae Ad.V relative to Ma.V (bone marrow without trabecular and cortical bones) was determined at 2-µm resolution in a 2mm (proximal) or 4mm (distal) region beginning right underneath the growth plate. Segmentation and morphological assessment of adipocytes was performed as previously described (33).

For bone volume fraction (BV/TV %) and Cortical thickness (Ct.th. mm) analyses, we used an in-house developed semi-automated protocol. Volumes of interest around the trabecular bone region were drawn in the proximal metaphysis starting directly underneath growth plate and covering a height of 1.2mm distal to the growth plate. Cortical thickness was determined in a mid-diaphyseal volume of interest starting 1mm proximal of the tibio-fibular junction, extending 1mm in the proximal direction.

### BMSCs culture

BMSCs were harvested from tibiae of 3-week-old *PiT2^-/-^* and *PiT2^+/+^* mice. After removing the epiphyses, bones were flushed to isolate total bone marrow cells and BMSCs were expanded in growth media consisting of α-MEM (Eurobio, France) supplemented with 15% fetal bovine serum (FBS), 100IU/mL penicillin, 100mg/mL streptomycin, 2mM glutamine. At confluence, BMSCs were cultured in adipogenic media consisting of α-MEM (Eurobio, France) supplemented with 15% FBS, 100IU/mL penicillin, 100mg/mL streptomycin, 2mM glutamine, 0.5µM 3-isobutyl-1-methylxanthine (IBMX), 0.5µM hydrocortisone and 60µM indomethacin (Sigma Aldrich).

### Oil Red O staining and relative quantification

Cells were fixed in 2% PFA for 15min, washed with water, incubated with 60% isopropanol for 5min and stained with newly filtered Oil Red O solution for 10min at room temperature. To quantify staining, Oil Red O was extracted from the cells with isopropanol and absorbance of the solution was measured at a wavelength of 520nm to determine the relative amount of dye. Determination of the number of adipocytes (Oil Red O positive cells) and the number of total cells (Hoechst positive cells) was performed by using the High Content Screening Arrayscan (ThermoScientific). Image acquisitions (361 images per well) were performed using the Cellomics ArrayScan VTI/HCS Reader (ThermoScientific) using x5 magnification. Images analysis was performed with CellProfiler (35).

### Flow cytometry

Flow cytometry was performed on a BD LSRFortessa™ (BD Biosciences), FACS data were collected using DIVA (Becton Dickinson) and analysed using FlowJo software (Tree Star). For flow cytometry analyses, BMSCs were isolated by flushing tibiae of P21 mice, flushed bones were crushed with a mortar and treated with collagenase for 20min at 37°C to retrieve any remaining BMSCs. The reaction was stopped by adding α-MEM complemented with 15% FBS and the cell suspension was passed through a 70µm cell strainer (BD Falcon, MA, USA) to remove bone fragments. The cells were then added to the previously flushed bone marrow with isolated BMSCs. Cells were centrifuged at 1200rpm for 5min at RT, the pellet was resuspended in Red Blood Cell Lysis Buffer (Sigma, Product No. R 7757) to eliminate red blood cells and centrifuged again at 1200rpm for 5min at RT. The pellet was resuspended and the cells were counted. Five million cells were stained with antibodies (Supporting **Table S1**) for 30min on ice. Compensation was performed using OneComp™ eBeads Compensation Beads (ThermoFisher Scientific Inc.). BMSCs were analysed as Ter119^-^ CD45^-^ Sca1^+^ CD44^+^ CD105^+^ (36, 37) and adipogenic progenitors as Ter119^-^ CD45^-^ CD31^-^ Sca1^+^ CD24^-^ (38).

### RNA isolation and RT-qPCR

Total RNA was prepared with TRIzol Reagent (ThermoFisher Scientific) according to the manufacturer’s instructions. The RNA was reverse transcribed and analysed on a Bio-Rad CFX96 detection system using SYBR Select Master Mix (Applied Biosystems, Warrington, UK). mRNA levels were normalized relative to beta-glucuronidase (*GusB*) and Pinin (*Pnn*) expression and quantified using the ΔΔCT method (39). RT-qPCR primers were: Pinin Forward (Fw)-ACCTGGAAGGGGCAGTCAGTA and Reverse (Rv)-ATCATCGTCTTCTGGGTCGCT, GusB Fw-CTCTGGTGGCCTTACCTGAT and Rv- CAGTTGTTGTCACCTTCACCTC, PiT1 Fw-TGTGGCAAATGGGCAGAAG and Rv-AGAAAGCAGCGGAGAGACGA, PiT2 Fw-CCATCGGCTTCTCACTCGT and Rv AAACCAGGAGGCGACAATCT, FABP4 Fw-GAATTCGATGAAATCACCGCA and Rv-CTCTTTATTGTGGTCGACTTTCCA, AdipoQ Fw- TCTCCTGTTCCTCTTAATCCTGCC and Rv-CATCTCCTTTCTCTCCCTTCTCTC.

### Statistics

Statistical analyses were performed using the GraphPad 8.0 software. Data were analysed for normal distribution within each experimental group using the Shapiro–Wilk normality test. Normally distributed data were analysed by ANOVA or t tests, as appropriate. Where data were not normally distributed, non-parametric tests were used. When appropriate, *p* values were adjusted for multiple comparisons as indicated in the figure legends. A *p* value of less than 0.05 was considered statistically significant (exact n and *p* values are indicated in the figures or legends). Data are expressed as means ± S.E.M. Units and abbreviations are reported in accordance with recently published guidelines for research relating to BM adiposity (40).

## Results

### Proximal BMAT volume does not increase in *PiT2^-/-^* mice between 3 and 16 weeks

**Figure 1:**
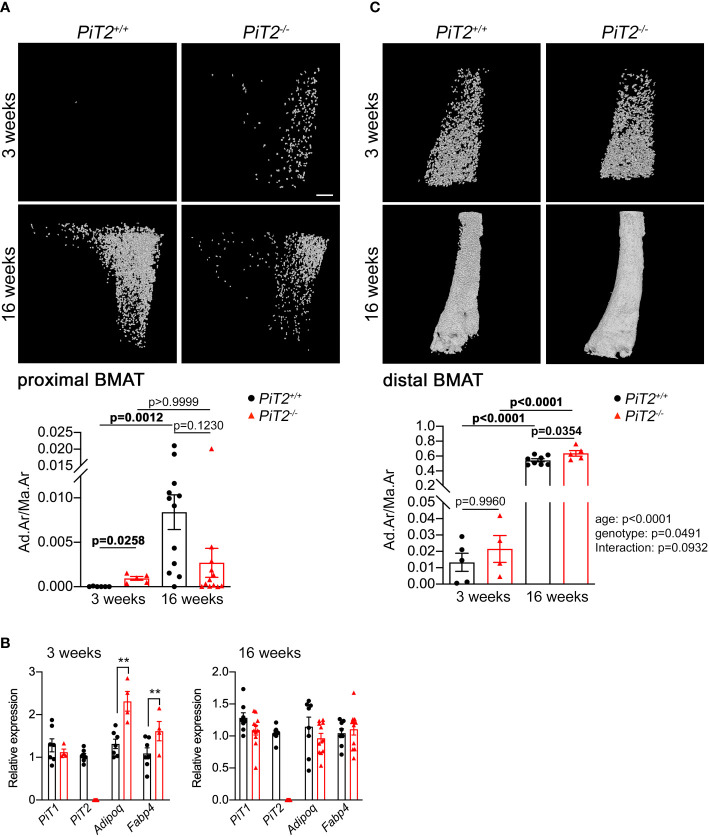
Deregulation of BMAT volume in *PiT2^-/-^* female mice. **(A)** *Upper -* Representative 3D visualization, using Hf-POM-based CE-CT, of the adipocytes in the bone marrow compartment of the proximal tibia of 3- and 16-week-old *PiT2^+/+^* and *PiT2^-/-^* female mice. Scale bar = 250μm. *Lower -* Graphs showing quantification of the volume fraction of the adipocytes in the VOI of the proximal tibia (Ad.Ar/Ma.Ar: Adipocyte area/Marrow Area) of 3- and 16-week-old *PiT2^+/+^* and *PiT2^-/-^* mice (n=6 and 12 per genotype, respectively). Data are means ± SEM, Bonferroni-corrected Mann-Whitney test. p values are indicated. **(B)** Relative *PiT1*, *PiT2*, *Adiponectin* and *FABP4* mRNA expression in the whole tibia from 3- (n=7 *PiT2^+/+^* and n=4-6 *PiT2^-/-^*) and 16- (n=8 *PiT2^+/+^* and n=11 *PiT2^-/-^*) week-old *PiT2^+/+^* and *PiT2^-/-^* female mice, as indicated. Data are means ± SEM, Mann-Whitney test, **p<0.01. **(C)** *Upper -* Representative 3D visualization of the adipocytes in the bone marrow compartment of the distal tibia of 3- and 16-week-old *PiT2^+/+^* and *PiT2^-/-^* female mice. *Lower -* Graphs showing quantification of the volume fraction of the adipocytes in the VOI of the distal tibia of 3- and 16-week-old *PiT2^+/+^* and *PiT2^-/-^* mice (n=6 and 11 per genotype, respectively). Data are means ± SEM, the influence of age or genotype, and interactions between these, was determined by two-way ANOVA, with *p* values shown to the right of the graph. p values from multiple comparisons are indicated on the graph.

Bone marrow adipose tissue was investigated by using Hf-POM-based CE-CT analysis on tibiae from 3- and 16-week-old *PiT2^+/+^* and *PiT2^-/-^* littermate mice. The 3D visualization and quantification of the bone marrow adipocytes showed an increase in BMAT volume within the proximal tibia of 3-week-old *PiT2^-/-^* female (**Figure 1A**, **Supplementary Figure 1**) and male (**Supplementary Figure 2A**, **Supplementary Figure 3**) mice compared to *PiT2^+/+^* mice, which is consistent with the increased mRNA gene expression of *Adiponectin* (*AdipoQ*) and *FABP4* (**Figure 1B**, **Supplementary Figure 2B**), two markers of mature adipocytes. The absence of *PiT2* mRNA expression was confirmed in whole tibia from *PiT2^-/-^* mice by RT-qPCR (**Figure 1B**, **Supplementary Figure 2B**). Interestingly, no difference in the expression of the paralog *PiT1* mRNA expression was observed in *PiT2^-/-^* whole tibia, making a possible PiT1-driven compensatory mechanism unlikely (**Figure 1B**, **Supplementary Figure 2B**).

In contrast to *PiT2^+/+^* mice, CE-CT analyses showed that BMAT volumes did not increase between 3 and 16 weeks in the proximal tibia of *PiT2^-/-^* mice, both in females (**Figure 1A**), and males, (**Supplementary Figure 2A**), leading to a downward trend in BMAT volume at 16 weeks in *PiT2^-/-^* compared to wild-type mice. This illustrates the possibility that the absence of PiT2 blunts the age-induced BMAT increase that is normally observed in the proximal tibiae of wild-type mice. Interestingly, in contrast to proximal tibiae, the increase in distal BMAT between 3 and 16 weeks was not affected by the absence of PiT2 at least in female (**Figure 1C**, not determined in males), suggesting a specific role for PiT2 in the proximal BMAT.

### Lack of PiT2 does not alter BMSCs adipogenic differentiation

**Figure 2:**
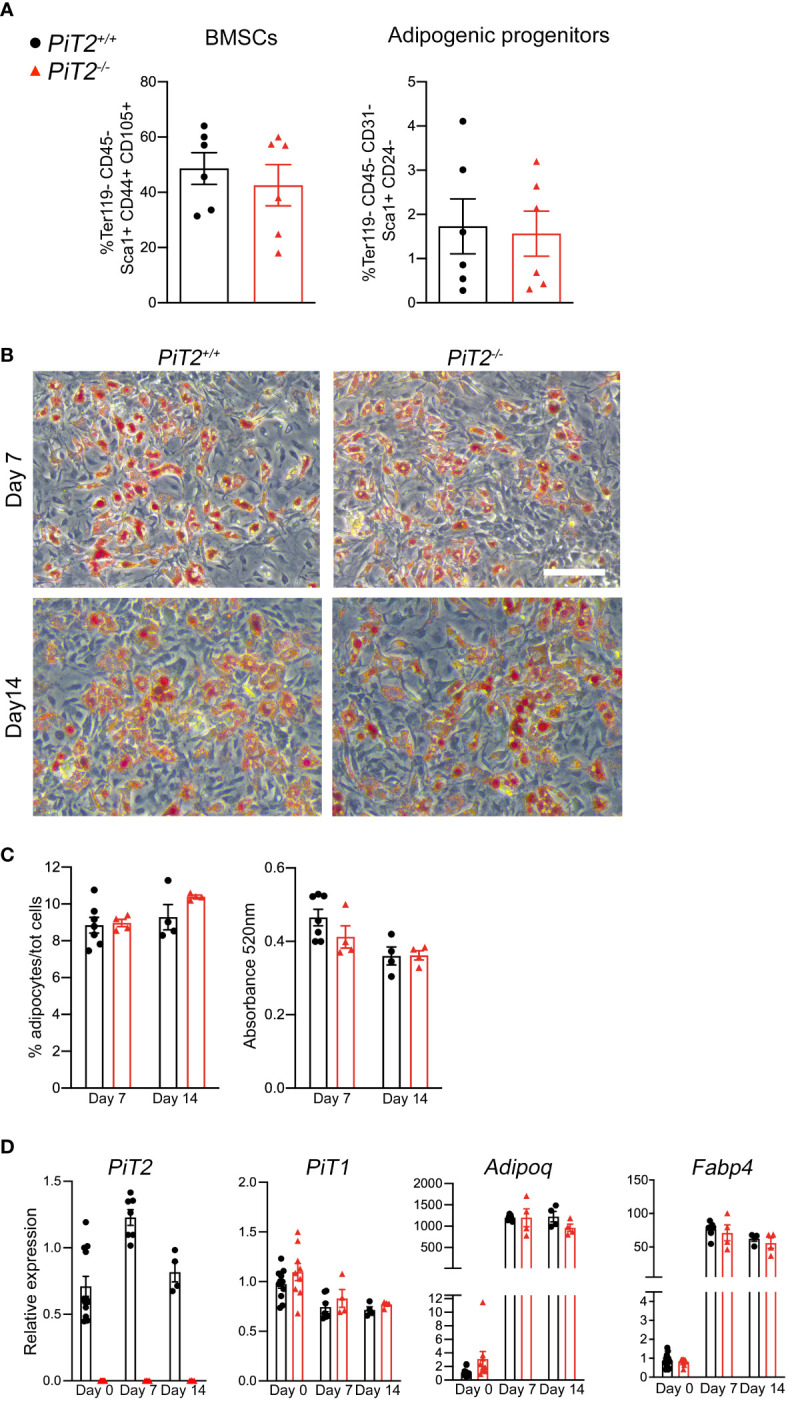
Characteristics of BMSCs in *PiT2^-/-^* mice. **(A)** FACS quantification of BMSCs (Ter119^-^ CD45^-^ Sca1^+^ CD44^+^ CD105^+^) (*Left*) and adipogenic progenitors (Ter119^-^ CD45^-^ CD31^-^ Sca1^+^ CD24^+^) (*Right*) in the bone marrow of tibiae from 3-week-old *PiT2^+/+^* and *PiT2^-/-^* female mice (n=6 per genotype). Data are means ± SEM, Mann-Whitney test. **(B)** Representative images of oil-red O (ORO) positive cells after adipogenic differentiation of *PiT2^+/+^* and *PiT2^-/-^* BMSCs. Scale bar = 100μm. **(C)** Quantitative analysis of ORO+ cells: *Left*, percentage of ORO+ cells related to the total number of Hoechst-positive cells; *right*, ORO absorbance at 520nm **(D)** Relative *PiT2*, *PiT1*, *Adiponectin* and *FABP4* mRNA expression at 0 (n=12 *PiT2^+/+^* and n=9 *PiT2^-/-^*), 7 (n=7 *PiT2^+/+^* and n=4 *PiT2^-/-^*) and 14 (n=4 *PiT2^+/+^* and n=4 *PiT2^-/-^*) days of differentiation. N=3 independent experiments. Data are means ± SEM. Two-way ANOVA test.

BMAds originate from the adipogenic differentiation of bone marrow stromal cells. To investigate the ability of PiT2-deficient BMSC to differentiate into adipocytes, we first quantified the adipogenic progenitors and explored the adipogenic differentiation capacity of BMSCs. We quantified these cells as Ter119^-^ CD45^-^ Sca1^+^ CD44^+^ CD105^+^ cells by flow cytometry. The results showed no difference in the proportion of BMSCs in 3-week-old *PiT2^-/-^* female samples compared to *PiT2^+/+^* mice (**Figure 2A**). Moreover, similar to BMSCs, analyses of adipogenic progenitors sorted as Ter119^-^ CD45^-^ CD31^-^ Sca1^+^ CD24^-^ cells, showed that the proportion of adipogenic progenitors in *PiT2^-/-^* female mice was equal to that of *PiT2^+/+^* mice (**Figure 2A**). Adipogenic differentiation analyses of BMSCs isolated from tibiae of 3-week-old *PiT2^-/-^* and *PiT2^+/+^* mice did not show differences between mutant and *PiT2^+/+^* cells from female and male mice, neither by quantification of Oil Red O positive cells nor by *Adiponectin* and *FABP4* mRNA expression analyses (**Figures 2B–D**, **Supplementary Figure 4**). Similar to what is observed *in vivo*, *PiT1* mRNA expression showed no differences between *PiT2^-/-^* and *PiT2^+/+^* cells (**Figure 2D** and **Supplementary Figure 4B**). Altogether, these results revealed that lack of PiT2 expression did not alter BMSCs populations or adipogenic differentiation, despite aberrant regulation of proximal BMAT in mutant mice.

### Regulation of bone and BMAT volumes in *PiT2^-/-^* mice after ovariectomy (OVX)

**Figure 3:**
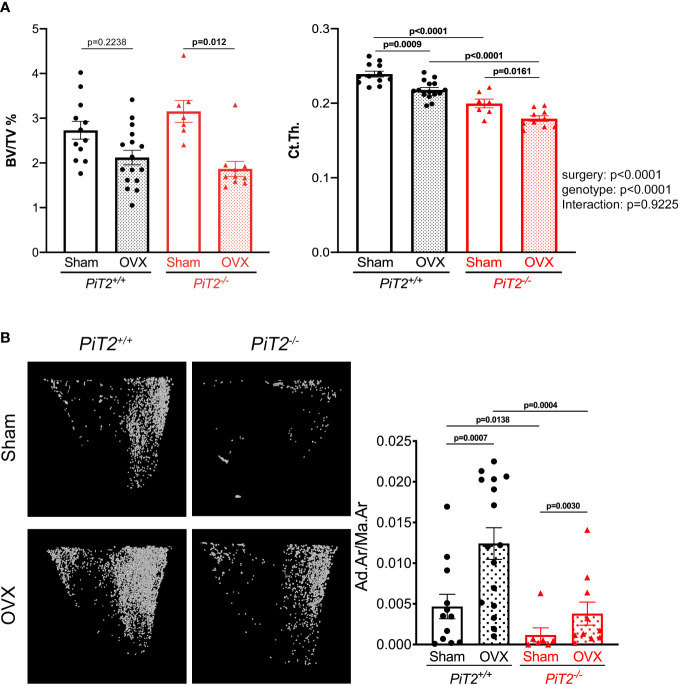
PiT2 deficiency does not prevent BMAT volume increase and bone mass loss after ovariectomy. **(A)** Bone volume/tissue volume (BV/TV, *left*) and cortical thickness (Ct.th., *right*) of tibia from shame operated mice (n=12 *PiT2^+/+^* and n=7 *PiT2^-/-^*) or after ovariectomy (OVX, n=16 *PiT2^+/+^* and n=10 *PiT2^-/-^*) quantified using CE-CT images. Data are means ± SEM, Bonferroni-corrected Mann-Whitney test (BV/TV) and two-way ANOVA test (Ct.Th.) For Ct.Th., the influence of surgery or genotype, and interactions between these, was determined by two-way ANOVA, with *p* values shown to the right of the graph. p values from multiple comparisons are indicated on the graphs **(B)** *Left:* representative 3D visualization, using Hf-POM-based CE-CT, of adipocytes in the bone marrow compartment of the proximal tibia (2mm height from the growth plate) of *PiT2^+/+^* and *PiT2^-/-^* female mice after ovariectomy (OVX). *Right*: graphs showing the quantification of the volume fraction of the adipocytes in the VOI of the proximal tibia (Ad.Ar/Ma.Ar: Adipocyte area/Marrow Area) in shamed operated mice (n=12 *PiT2^+/+^* and n=7 *PiT2^-/-^*) or after ovariectomy (OVX, n=16 *PiT2^+/+^* and n=10 *PiT2^-/-^*). Data are means ± SEM, Bonferroni-corrected Mann-Whitney test.

To evaluate the regulation of bone and BMAT volumes, 13-week-old *PiT2^-/-^* female mice and their control littermate (*PiT2^+/+^*) were subjected to OVX-induced osteoporosis. Five weeks after surgery, animals were analyzed for bone and BMAT volumes by CE-CT. Results show that sham-operated *PiT2^-/-^* female mice have similar trabecular BV/TV as *PiT2^+/+^* control mice, while cortical thickness was significantly reduced (**Figure 3A**), consistent with our previous study (31). In OVX mice, we showed a similar reduction in trabecular BV/TV and Ct.Th. in wild-type and *PiT2^-/-^* mice (**Figure 3A**). This reduction in bone volume parallels an increase in BMAT volume observed both in ovariectomized *PiT2^+/+^* and *PiT2^-/-^* mice compared with sham-operated controls (fold-change: 2.65 and 3.22, respectively; **Figure 3B**). Of note, sham-operated *PiT2^-/-^* mice showed a reduced BMAT volume compared with sham-operated *PiT2^+/+^* mice (**Figure 3B**), consistent with our observation in 16-week-old females (**Figure 1A**). These findings indicate that bone and BMAT volumes are inversely regulated in the model of ovariectomy-induced bone loss, and suggest that PiT2-deficient adipocytes retain the ability to be recruited after OVX.

## Discussion

In the present study, we report for the first time a role for PiT2 in the maintenance of bone marrow adipose tissue volume. We also show that BMSCs with or without PiT2 are able to differentiate into adipocytes in an identical manner, suggesting that the regulation of proximal BMAT volume by PiT2 does not involve cell autonomous mechanisms.

### Increased BMAT volume in young PiT2^-/-^ mice

An increase in marrow adipocytes which inversely parallels a loss of bone volume is observed in numerous genetic models (6, 8, 10). This link between bone and BMAT volumes cross-regulation is based on either the balance between osteo-adipogenic differentiation of BMSC or the secreted factors from bone cells or BMAds which can act on the surrounding tissue (6, 8, 10, 41). Our previous work showed that bone mass in young (3-week-old) *PiT2^-/-^* mice was reduced compared to *PiT2^+/+^* mice (31), and we now show, conversely, that proximal BMAT volume is higher compared with wild-type mice. Although this is consistent with the expected inverse cross-regulation between bone and adipocyte volumes, we nevertheless show that adipogenic differentiation of PiT2-deficient BMSCs is not altered. However, the *in vitro* adipogenesis conditions used in our study may be so potent that they override any subtle differences in BMSC adipogenic potential that exist *in vivo*. In addition, the use of more sensitive technologies, such as single cell RNA sequencing, the use of other recently identified adipogenic progenitor markers could help to identify other *in vivo* stromal populations that modulate adipogenesis and whose expression may be abnormal in *PiT2^-/-^* mice (42–45). To date we have not identified which cellular or molecular mechanism affected by the loss of PiT2 deregulates adipocyte volume control. However, we interestingly observed that proximal BMAds can be detected at least from 16 days of age (not shown) in the *PiT2^-/-^* mice, which is very early compared to the appearance of proximal BMAT in wild-type mice that can only be observed from 4 weeks of age in C57Bl/6 (46). This may therefore suggest that PiT2 deletion affects cellular or molecular pathways that occur during development or very early after birth and may provide an invaluable study model to identify them.

### No inverse correlation between BMAT and bone volumes in adult PiT2^-/-^ mice

In adult *PiT2^-/-^* mice, the reduced bone mass is not associated with an increased BMAT volume compared to *PiT2^+/+^* mice. As we have shown previously, the bone defects in mutant mice are independent of osteoblast or osteoclast number or activity (31), supporting the hypothesis that BMAT and bone defects in the absence of PiT2 are not generated by a coupling imbalance between adipocytes and osteoblasts differentiation.

BMAT volume is known to be regulated by several hormones (*e.g.*, PTH, estrogen or FSH) (2, 3). However, we have previously shown that serum PTH levels were similar between *PiT2^+/+^* and *PiT2^-/-^* mice, excluding its role in the genesis of the phenotype (47). In post-menopausal women, BMAT volume is highly increased and ovariectomy is now a commonly used approach to increase BMAT volume in animal models (7, 10, 34, 48, 49). Estrogen, in addition to be a negative regulator of BMAds, is a positive regulator of the bone mass (48). These opposite effects of estrogen on bone and fat tissues may be explained by the imbalanced ability of BMSCs to differentiate into osteoblasts in favor of adipocytes, although no direct evidence has yet been obtained. In *PiT2^-/-^* mice, both bone and BMAT volumes are reduced and a similar phenotype is observed in males, ruling out the possibility that a defect in estrogen levels could explain the phenotype of these mutant mice. Hence, further studies will be needed to identify the possible systemic factor underlying the alterations in bone and marrow adipose tissues.

### Bone and bone marrow adipose tissues are sensitive to estrogen depletion in PiT2^-/-^ mice

The observation that BMAT volume does not increase between 3 and 16 weeks in *PiT2^-/-^* mice led us to question the regulation of bone and adipose tissue volumes in pathological conditions. In ovariectomized *PiT2^-/-^* mice, we showed that bone mass is decreased and that this is accompanied by BMAT accumulation similar to that in control mice. These results suggest that in the absence of PiT2 adipogenic differentiation is not affected and that the mechanisms that govern it are upstream of PiT2-dependent pathways. On the other hand, it is well documented that OVX-induced bone loss is primarily caused by induction of bone resorption. Here, we show that bone loss is similar in OVX- *PiT2^+/+^* mice and OVX-*PiT2^-/-^* mice, indicating that osteoclast formation and activity are not impaired, consistent with our previous findings that showed normal osteoclast activity in *PiT2^-/-^* mice (31). The lack of effect of PiT2 deletion on bone and fat phenotypes during ovariectomy is consistent with the fact that we do not observe differences in male and female mutant mice. As these results were obtained in adult mice, they may also support the idea that the role of PiT2 in the bone and BMAT phenotypic balance occurs earlier in the development or during the growth of the animal.

### Could phosphate homeostasis explain the BMAT phenotype in PiT2^-/-^ mice?

As one of the major cellular functions of PiT2 is to transport phosphate across the plasma membrane, it is reasonable to ask whether the observed phenotype of *PiT2^-/-^* mice may be phosphate-related. In the literature, a link between phosphate and adipose tissues is rarely reported, either in extra- or intra-medullar adipose tissues. In humans, an inverse correlation between serum Pi levels and obesity has been observed, and an association between high phosphate diet and the suppression of lipogenesis in white adipose tissue has been described both in humans and rodents (50–57). Interestingly, acute phosphate restriction in mice decreases bone formation and results in an increase in BMAT due to defects in the commitment of BMSC preferentially towards the adipogenic lineage involving the Wnt signaling pathway (58, 59). It is therefore possible that a change in phosphate homeostasis may impact the balance between bone formation and BMAT. However, *PiT2^-/-^* mice show no differences in serum phosphate levels and key markers of phosphate homeostasis regulation (31, 32, 60, 61), precluding a role for serum phosphate in the BMAT defects observed in the absence of PiT2. However, as we hypothesize for the dental defects in the *PiT2^-/-^* mice (62), the local extracellular Pi level, rather than the serum Pi levels, may be responsible for the observed phenotype. Interestingly, invalidation of *Phospho1*, another phosphate-related gene, results in a reduction in bone volume and bone mineral density, along with an increase in BMAT volume (63, 64). Whether this phenotype is directly due to an abnormal local level of Pi within the skeleton or whether it is an indirect consequence of modified energy metabolism remains to be determined.

In summary, we have shown that loss of PiT2 leads to defects in bone and marrow adipose tissues, probably through a non-intrinsic defect of osteoblasts, osteoclasts and BMAds, leading to impaired bone quality and strength. Further identification is needed to decipher the underlying cellular mechanisms.

## Data availability statement

The original contributions presented in the study are included in the article/**Supplementary Materials**. Further inquiries can be directed to the corresponding author.

## Ethics statement

Experiments on mice were conducted according to the French and European regulations on care and protection of laboratory animals (EC Directive 86/609, French Law 2001-486 issued on June 6, 2001). This study complied with ARRIVE (Animal Research: Reporting of In Vivo Experiments) guidelines and was approved by the Animal Care Committee of Pays de la Loire (APAFIS agreements 02286.02, 14835-2020-05-13 and 22178-2019092614593965 v7).

## Author contributions

Study design: GF, LB, and SB-C. Data collection: GF, MG, GP, PG, GK, MF, JV, BH, SS and SB-C. Data analysis and interpretation: GF, MG, GP, GK, MF, XP, LB and SBC. Drafting manuscript: GF, LB and SB-C. Revising manuscript content: GF, MG, JG, XP, LB and SB-C. Approving final version of manuscript: all authors. SBC takes responsibility for the integrity of the data analysis.

## Funding

This work was supported by grants from Inserm, Region des Pays de la Loire (AdipOs) and Société Française de Rhumatologie (PITAMO and METABONE). GF and MG received a doctoral fellowship from Nantes Université.

## Acknowledgments

The authors are grateful to the Experimental Health core facility (PES) and SC3M histology facility of the SFR Bonamy (Nantes, France) for their technical support, and MicroPiCell and Cytocell facilities (UMS BioCore, Inserm US16/CNRS UAR3556) for expert technical assistance. The authors gratefully acknowledge Carla Geeroms (Department of Materials Engineering, KU Leuven, Leuven, BELGIUM), Céline Menguy (Inserm U1087, Nantes, France) and Séverine Marionneau (Inserm U1232, Nantes) for technical help. The authors also gratefully acknowledge Dr Christophe Chauveau (MABLab, Boulogne-sur-mer, France) and Dr Céline Colnot (IMRB, Paris) for helpful discussions.

## Conflict of interest

The authors declare that the research was conducted in the absence of any commercial or financial relationships that could be construed as a potential conflict of interest.

## Publisher’s note

All claims expressed in this article are solely those of the authors and do not necessarily represent those of their affiliated organizations, or those of the publisher, the editors and the reviewers. Any product that may be evaluated in this article, or claim that may be made by its manufacturer, is not guaranteed or endorsed by the publisher.

## Supplementary material

Supplementary Figure 1 Three-D visualization, using Hf-POM-based CE-CT, of the adipocytes in the bone marrow compartment of the proximal tibia of 3-week-old *PiT2^+/+^* and *PiT2^-/-^* female mice.

Supplementary Figure 2 Deregulation of BMAT volume in *PiT2^-/-^* male mice. **(A)** *Upper -* Representative 3D visualization, using Hf-POM-based CE-CT, of the adipocytes in the bone marrow compartment of the proximal tibia (2mm height from the growth plate) of 3- and 16-week-old *PiT2^+/+^* and *PiT2^-/-^* male mice. *Lower -* Graph showing quantification of the volume fraction of the adipocytes in the proximal tibia *PiT2^+/+^* and *PiT2^-/-^* mice at 3 weeks (n=5 and 3, respectively) and 16 weeks (n=6 and 5, respectively). Data are means ± SEM, Bonferroni-corrected Mann-Whitney test. (B) Relative *PiT1*, *PiT2*, *Adiponectin* and *FABP4* mRNA expression in the whole tibia from 3- (n=5 per genotype) and 16- (n= 7 *PiT2^+/+^* and n= 5 *PiT2^-/-^*) week-old *PiT2^+/+^* and *PiT2^-/-^* male mice, as indicated. Data are means ± SEM, Mann-Whitney test, **p<0.05*.

Supplementary Figure 3 Three-D visualization, using Hf-POM-based CE-CT, of the adipocytes in the bone marrow compartment of the proximal tibia of 3-week-old *PiT2^+/+^* and *PiT2^-/-^* male mice.

Supplementary Figure 4 Adipogenic differentiation of BMSCs from *PiT2^-/-^* males. **(A)** *Left -* Representative images of *PiT2^+/+^* and *PiT2^-/-^* BMSCs after adipogenic differentiation and ORO staining. Scale bar = 100μm. *Right -* Graphs showing the percentage of oil-red O (ORO) positive cells related to the total number of Hoechst-positive cells (left) and ORO absorbance at 520nm (right). **(B)** Relative *PiT1*, *Adiponectin* and *FABP4* mRNA expression at 0 and 7 days of differentiation, n are indicated on graph bars. Data are means ± SEM, Mann-Whitney test.
