# Supplementary figures and images for: PiT2 deficiency prevents increase of bone marrow adipose tissue during skeletal maturation but not in OVX-induced osteoporosis
Source: Front Endocrinol (Lausanne). 2022 Nov 16;13:921073. doi: 10.3389/fendo.2022.921073 (PMC9708882; doi:10.3389/fendo.2022.921073)

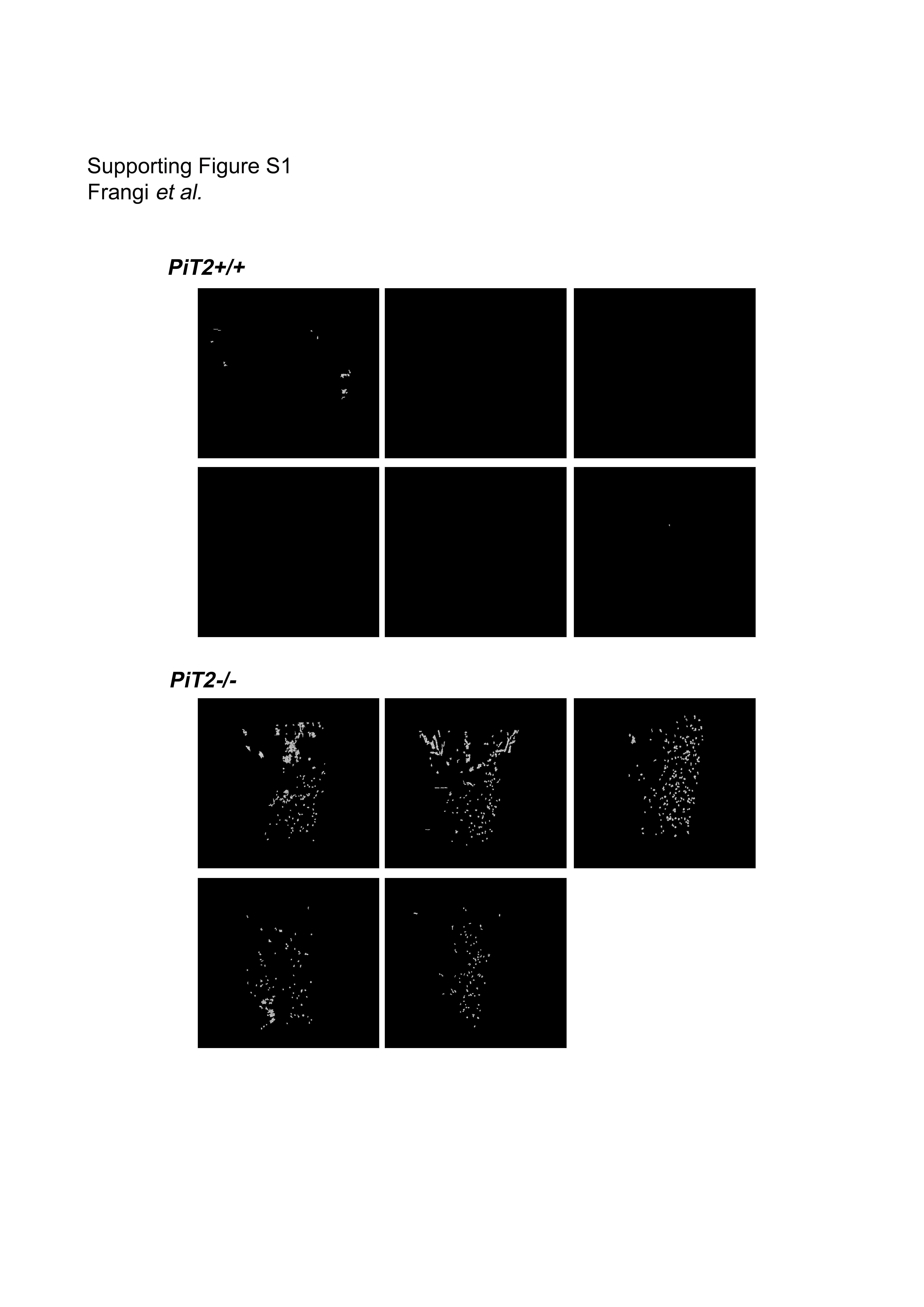

Supplement: Supplementary Figure 1 — Three-D visualization, using Hf-POM-based CE-CT, of the adipocytes in the bone marrow compartment of the proximal tibia of 3-week-old PiT2+/+ and PiT2-/- female mice. [file Image_1.tif]

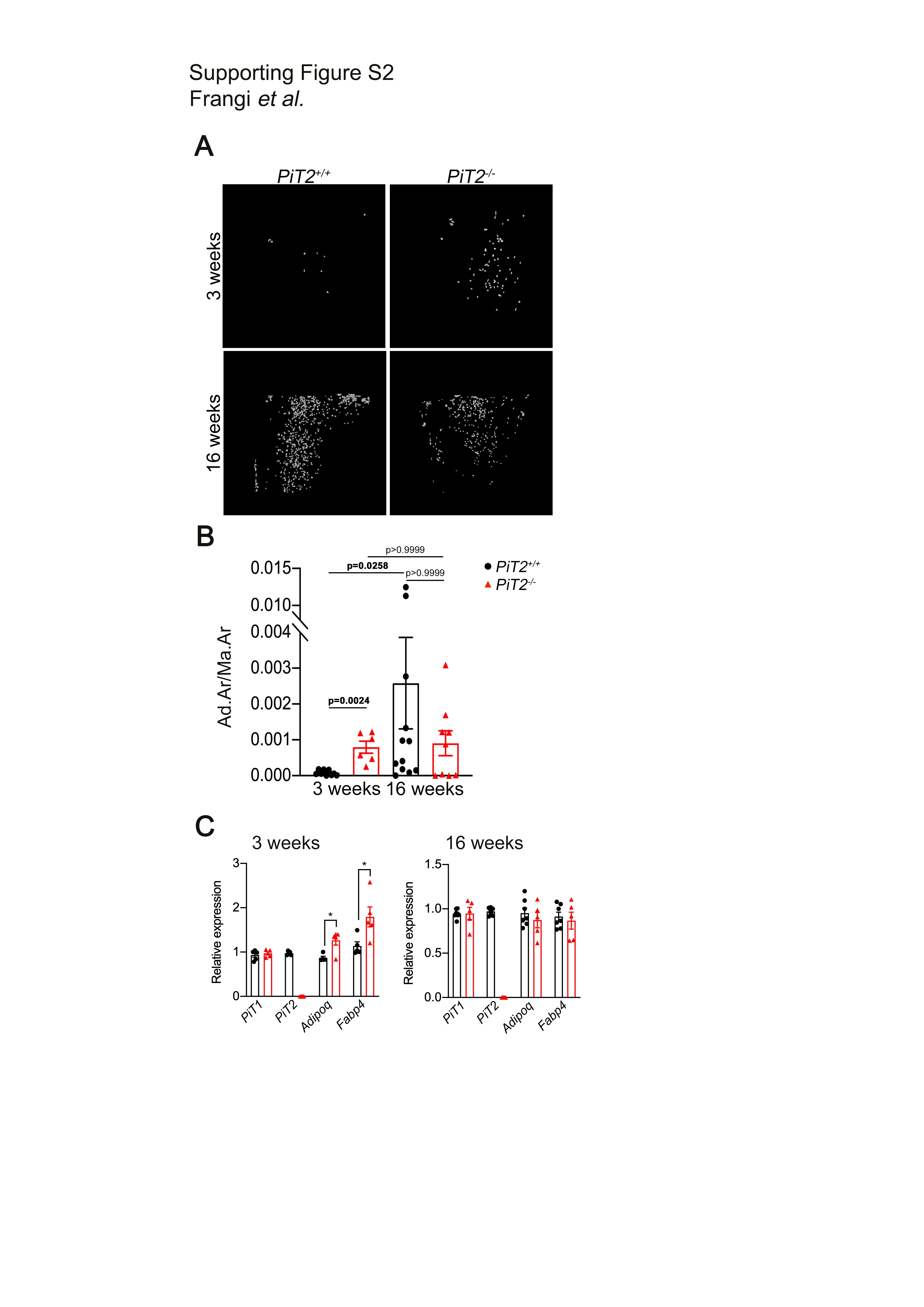

Supplement: Supplementary Figure 2 — Deregulation of BMAT volume in PiT2-/- male mice. (A) Upper - Representative 3D visualization, using Hf-POM-based CE-CT, of the adipocytes in the bone marrow compartment of the proximal tibia (2mm height from the growth plate) of 3- and 16-week-old PiT2+/+ and PiT2-/- male mice. Lower - Graph showing quantification of the volume fraction of the adipocytes in the proximal tibia PiT2+/+ and PiT2-/- mice at 3 weeks (n=5 and 3, respectively) and 16 weeks (n=6 and 5, respectively). Data are means ± SEM, Bonferroni-corrected Mann-Whitney test. (B) Relative PiT1, PiT2, Adiponectin and FABP4 mRNA expression in the whole tibia from 3- (n=5 per genotype) and 16- (n= 7 PiT2+/+ and n= 5 PiT2-/- ) week-old PiT2+/+ and PiT2-/- male mice, as indicated. Data are means ± SEM, Mann-Whitney test, *p<0.05. [file Image_2.tif]

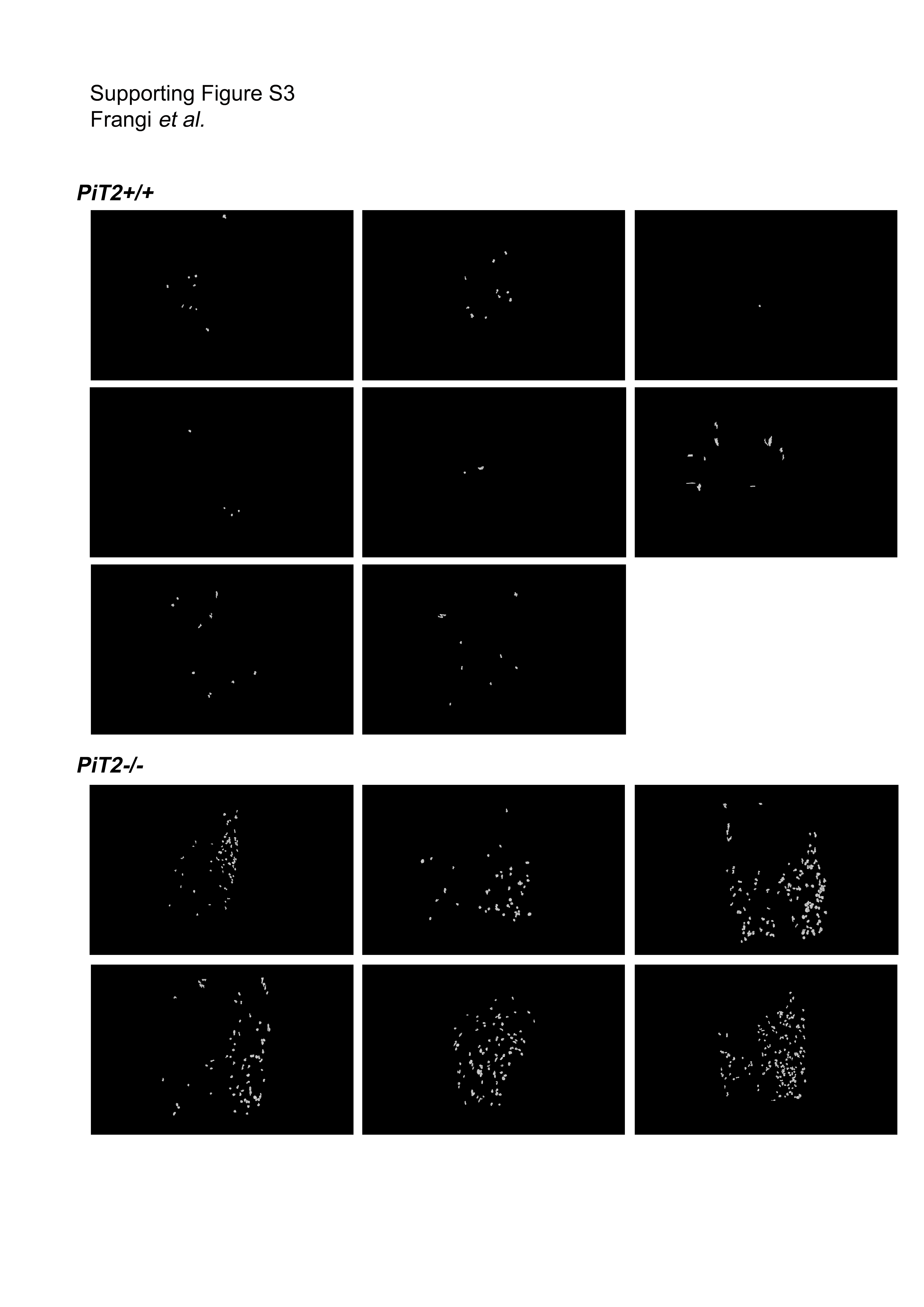

Supplement: Supplementary Figure 3 — Three-D visualization, using Hf-POM-based CE-CT, of the adipocytes in the bone marrow compartment of the proximal tibia of 3-week-old PiT2+/+ and PiT2-/- male mice. [file Image_3.tif]

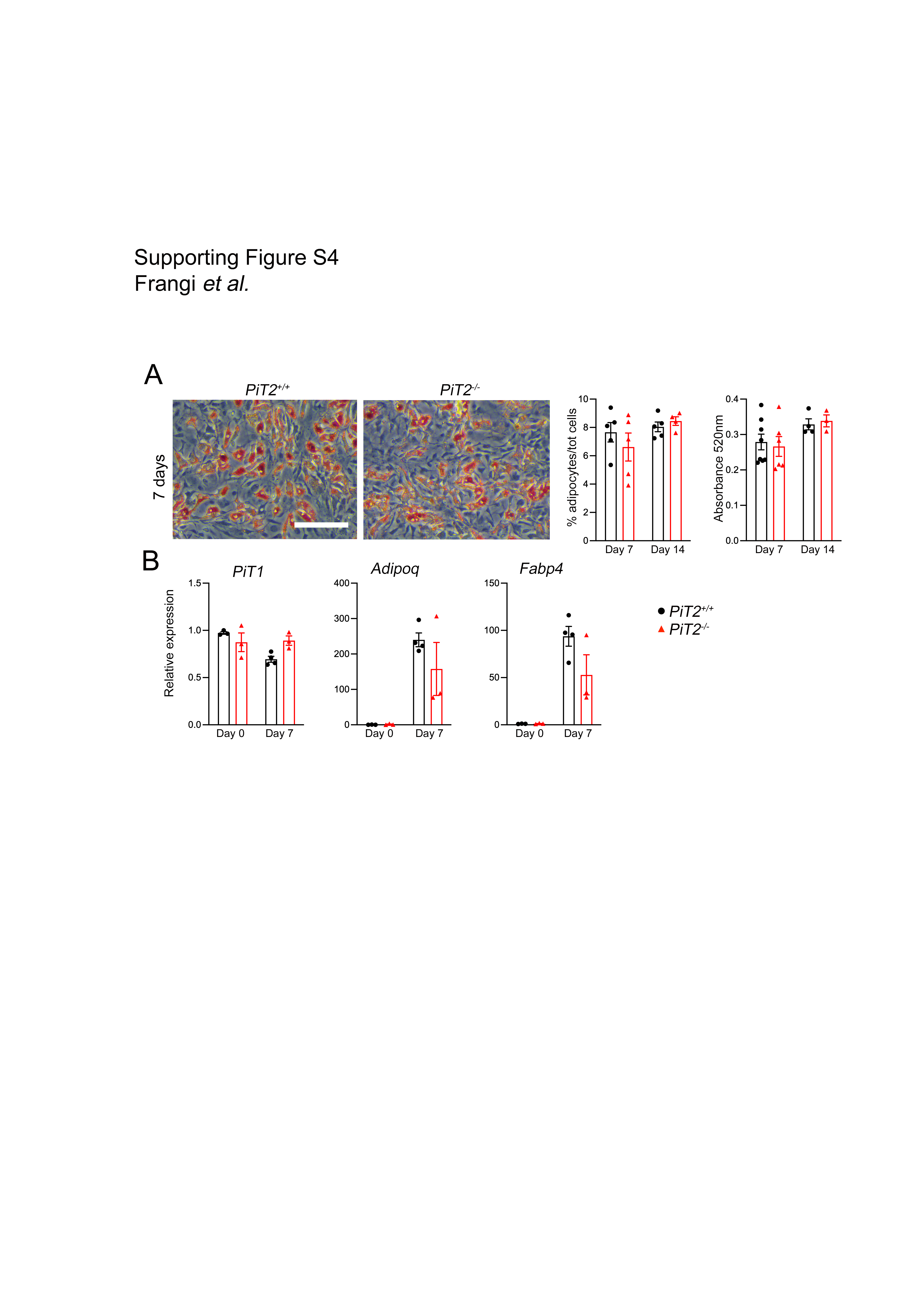

Supplement: Supplementary Figure 4 — Adipogenic differentiation of BMSCs from PiT2-/- males. (A) Left - Representative images of PiT2+/+ and PiT2-/- BMSCs after adipogenic differentiation and ORO staining. Scale bar = 100μm. Right - Graphs showing the percentage of oil-red O (ORO) positive cells related to the total number of Hoechst-positive cells (left) and ORO absorbance at 520nm (right). (B) Relative PiT1, Adiponectin and FABP4 mRNA expression at 0 and 7 days of differentiation, n are indicated on graph bars. Data are means ± SEM, Mann-Whitney test. [file Image_4.tif]
