# Supplementary material for: PiT2 deficiency prevents increase of bone marrow adipose tissue during skeletal maturation but not in OVX-induced osteoporosis
Source: Front Endocrinol (Lausanne). 2022 Nov 16;13:921073. doi: 10.3389/fendo.2022.921073 (PMC9708882; doi:10.3389/fendo.2022.921073)
Supplement: Supplementary file 5 [file Table_1.docx]

**Table S1.** Antibodies used for cytometry analysis

| **Antibodies:** | **Source:** | **Identifier:** |
| --- | --- | --- |
| APC Rat Anti-Mouse CD31 | BD Biosciensces | Cat#: 551262 |
| BV510 Rat Anti-Mouse TER-119 | BD Biosciensces | Cat#: 563995 |
| BV510 Rat Anti-Mouse CD45 | BD Biosciensces | Cat#: 563891 |
| APC Rat Anti-Mouse CD44 | BD Biosciensces | Cat#: 559250 |
| BV421 Rat Anti-Mouse Ly-6A/E | BD Biosciensces | Cat#: 562729 |
| PE Rat Anti-Mouse CD24 | BD Biosciensces | Cat#: 561079 |
| BV650 Rat Anti-Mouse CD105 | BD Biosciensces | Cat#: 740609 |
